# Paclitaxel and cisplatin combined with intensity-modulated radiotherapy for upper esophageal carcinoma

**DOI:** 10.1186/1748-717X-8-75

**Published:** 2013-03-27

**Authors:** Lingli Tu, Lan Sun, Yong Xu, Yongsheng Wang, Lin Zhou, Yongmei Liu, Jiang Zhu, Feng Peng, Yuquan Wei, Youling Gong

**Affiliations:** 1 Department of Thoracic Oncology and State Key Laboratory of Biotherapy, Cancer Center, West China Hospital, Sichuan University, Chengdu, 610041, People’s Republic of China

**Keywords:** Upper esophageal carcinoma, Paclitaxel, Cisplatin, Intensity-modulated radiotherapy

## Abstract

**Purpose:**

This study was conducted to evaluate the effectiveness and safety of intensity-modulated radiotherapy (IMRT) and concurrent paclitaxel plus cisplatin (TP regimen) for upper esophageal carcinoma.

**Methods:**

36 patients of upper esophageal carcinoma were retrospectively analyzed. Patients were treated with IMRT (median 60 Gy) combined with concurrent TP regimen chemotherapy. The Kaplan-Meier analysis was performed in statistical analysis. Toxicities were recorded according to the NCI CTC version 3.0.

**Results:**

36 patients aged 43–73 years (median 57 years). The median follow-up period was 14.0 months. The 1-year and 2-year survival rates were 83.3% and 42.8% respectively. The median progression-free survival (PFS) time and overall survival (OS) time were 12.0 (95% CI: 7.8–16.2 months) and 18.0 months (95% CI: 9.9–26.1 months), respectively. Grade 3 neutropenia, radiation-induced esophagitis and radiodermatitis were observed in 5 (13.9%), 3 (8.3%) and 8 (22.2%) patients respectively. There were two treatment-related deaths due to esophageal perforation and hemorrhea.

**Conclusions:**

For those patients with upper esophageal carcinoma, IMRT combined with concurrent TP regimen chemotherapy was an effective treatment. However, more attention should be paid to the occurrence of perforation and hemorrhea.

## Introduction

The incidence of esophageal carcinoma is increasing in the world as well as China. About 462,000 cases of newly esophageal carcinoma were diagnosed worldwide every year. It is the sixth leading cause of death from cancer, and the overall 5-year survival rate is only 10% [1,2]. Upper esophageal carcinoma, including cervical and upper thoracic region, is relatively uncommon and accounts for only 5%–10% of all esophageal carcinomas [3]. However, it has a poor prognosis, and the reported 3- and 5-year survival rates with surgical resection range from 18% to 35.4% and from 12% to 33%, respectively [4].

Surgery remained the gold standard of curative treatment for carcinoma of esophagus. However, carcinoma of upper esophagus was difficult to be resected and achieved a clear margin based on complicated anatomic structure. Besides, surgical complication and mortality rates were severe, and the 5-year survival rate after surgery was only 14–16% [5,6]. Chiu *et al.* conducted a prospective randomized trial to compare standard esophagectomy with definitive chemo-radiotherapy (CRT) for patients with potentially resectable squamous cell carcinoma of esophagus [7]. Two groups achieved a similar disease-free survival (24 *vs.* 20 months) and overall survival (24 *vs.* 21 months). Therefore, the definitive radiochemotherapy is generally considered as the standard treatment for upper esophageal carcinoma.

Currently, the optimal therapeutic schedule of upper esophageal carcinoma remains undetermined, although the 5-fluorouracil (5-FU) plus cisplatin (DDP) combined with radiotherapy was generally recognized as the initial strategy. The regarding data on other chemotherapeutics in patients of upper esophageal carcinoma has been lacking. Paclitaxel, a new broad-spectrum cytotoxic antineoplastic, has shown some promising responses against a great many carcinomas. As a single agent, paclitaxel has been shown to have a response rate of 32% in esophageal cancer [8]. In addition, several Phase II studies have found that paclitaxel-based regimens have significant activity in patients with locally advanced and metastatic esophageal cancer [9-11]. It had been also demonstrated *in vitro* that paclitaxel had radioenhancing effects in some tumor cell lines [12-14]. And the combination of paclitaxel and platinum with concurrent radiotherapy really showed a good response in patients with esophageal cancer [15,16].

It is a challenge to deal with the target conformity and risk organ sparing with 3-dimentional conformal radiotherapy (3D-CRT) in treating upper esophageal carcinoma. Intensity-modulated radiation therapy (IMRT) represents a fundamentally new approach to the planning and delivery of radiation therapy. It combines two advanced concepts to deliver 3D-CRT: inverse treatment planning with computerized optimization and computer-controlled intensity modulation of the treatment beams, demonstrating the dosimetric superiority over 3D-CRT approaches in nearly all of the major tumor sites.

So far, a few studies reported the concurrent CRT for upper esophageal carcinoma [17-19], and the chemotherapy they applied was the 5-FU based regimen. To our knowledge, no data had been reported regarding to the combination of TP regimen and IMRT technique. Thus we conducted a retrospective study to evaluate the effectiveness and safety of IMRT and concurrent TP regimen for upper esophageal carcinoma.

### Patients and methods

#### Patients’ data

From August 2006 to November 2011, all patients of upper esophageal carcinoma treated with concurrent chemoradiotherapy without surgery in West China Hospital were retrospectively analyzed. To be included in our analysis, patients needed to meet the following criteria: All patients had a histologically proven esophageal carcinoma; tumor was located in the cervical or thoracic upper esophagus without visceral metastasis by esophagogastroduodenoscopy, esophagography and computed tomography (CT scan) at the time of diagnosis; they were firstly treated with IMRT and concurrent TP regimen without surgery. Besides, we excluded patients if they had treatment with radiotherapy alone, unaccomplished radiotherapy, recurrent disease, or the other tumors in middle or lower esophagus.

#### Tumor evaluation

Tumor evaluation was based on esophagogastroduodenoscopy, esophagography, neck/chest/abdominal CT, and endoscopic ultrasound of the esophagus. Tumor baseline characteristics (TNM stage, location, size, and histopathology) were taken. The tumor staging was based on the 2002 American Joint Committee on Cancer (AJCC) staging system [20]. The tumor length was defined by esophagogastroduodenoscopy or/and barium esophagography and tumor diameter by CT scan. The upper esophageal carcinoma was located in esophagus above tracheal eminence, and 24 cm from incisor tooth by esophagogastroduodenoscopy.

#### IMRT

Each patient was immobilized in the supine position. The planning CT scans were performed at 3 mm slice thickness using a dedicated helical CT scanner (Siemens, Somatom Plus^4^) throughout the entire neck and thorax. All of the CT images of patients acquired were transferred to and registered in the treatment planning system (TPS) with the same method. The gross tumor volume (GTV) included all macroscopic tumors and enlarged lymph nodes as determined by the imaging and endoscopic findings. The clinical target volume (CTV) was defined as the GTV plus a 2–3 cm radial margin. If the target was contoured in the supraclavicular region, the correlated lymphatic drainage region was contoured as the CTV, extending to the cricothyroid membrane. The planning target volume (PTV) was defined as the CTV plus a 0.5 cm margin in all direction, respectively. The median irradiation dose for the PTV was 60 Gy, with a range of 52–70 Gy at 1.8–2.0 Gy per fraction and 5 fractions per week. The prescription dose covered at least 95% of the volume of the PTV and the hot point was limited within the 107% of the prescription dose. The dose constraint for the spinal cord was a maximum dose < 45 Gy. For lungs, the mean dose and V20 were limited within 15 Gy and 30% respectively. The IMRT plans were generated using 5 or 7 co-planar beams with a 6-MV linear accelerator.

#### Chemotherapy

The concurrent chemotherapy regimen started at the first day of radiotherapy. The regimens consisted of paclitaxel 135 mg/m^2^ and DDP 75 mg/m^2^ on day one per 3 weeks. If the grade 3 or higher treatment-related esophagitis were found and lasting, the chemotherapy would be suspended until recovery and reduced sequentially the regimen dose by 25% in the subsequent cycle. All toxicities related to the treatment were evaluated using the National Cancer Institute Common Toxicity Criteria (NCI CTC, version 3.0).

If grade 3 or higher side-effects were observed, the nutritive sucking during gavage feedings and symptomatic management was added during the treatment.

#### Response to CRT and follow-up

Evaluation of treatment response was carried out according to Response Evaluation Criteria in Solid Tumors (RECIST criteria) [21]. The evaluation was performed 1 month after CRT completion. The follow-up was performed on a clinical basis, with barium esophagography and chest and abdominal CT scans every 3 months for first year and every 6 months thereafter. Follow-up data were updated in May 2012.

#### Statistical analysis

Overall survival (OS) was calculated from the date of CRT initiation until the date of death or the date of last follow-up. Survival curve was established using Kaplan-Meier method. Progression-free survival (PFS) was estimated from the date of the first day of CRT initiation to the time of documented failure (local recurrence or metastasis occurrence) or the date of the last follow-up for those remaining with CCR. A value of *p* < 0.05 (2-sided) was considered with statistical significance. Statistical analysis was performed using SPSS 13.0 software.

## Results

As shown in Table 1, 36 patients were evaluated in this analysis. All cases were squamous cell carcinoma. The median follow-up periods for 31 patients was 14.0 months (range: 5.0- 65.0 months), 5 cases (13.9%) had been lost to follow-up. All patients completed the radiotherapy treatment. 83.3% (30/36) patients had received 2 cycles of TP chemotherapy, while the remaining patients had received at least 1 cycle of chemotherapy.

**Table 1:** Clinical characteristics of the patients (n = 36)

**Characteristics**	**Number of patients (%)**
***Age (years)***	
Median (range)	57 (43–73)
< 65 years	27 (75)
> 65 years	9 (25)
***Gender***	
Male/Female	31 (86.1)/5 (13.9)
***ECOG***^***a ***^***performance status***	
0-1	32 (94.4)
2	4 (5.6)
***Location***	
Cervical esophagus	4 (5.6)
Upper thoracic esophagus	32 (94.4)
***Tumor length***	
< 5 cm	16 (44.4)
> 5 cm	20 (55.6)
***Clinical tumorstage***^***b***^	
II stage	11 (30.6)
III stage	13 (36.1)
IV stage	12 (33.3)

^*a*^: Eastern Cooperative Oncology Group; ^*b*^: Staging system, 6^th^ edition, American Joint Committee on Cancer, 2002.

### Responses to treatment

All patients were assessed as having had a response (Table 2). 6 (16.7%), 12 (33.3%) and 15 (41.7%) patients showed complete response (CR), partial response (PR) and stable disease (SD), respectively. The overall responses were 50% (18/36).

**Table 2:** Response to treatment

	**Complete response (CR)**	**Partial response (PR)**	**Stable disease (SD)**	**Progression disease (PD)**
**TP + R**	6 (16.7%)	12 (33.3%)	15 (41.7%)	3 (8.3%)

### Follow-up

**Figure 1:**
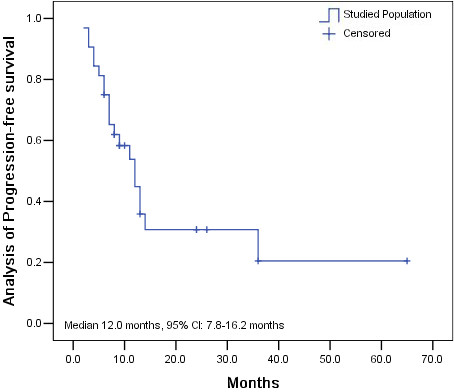
Analysis of progression-free survival (PFS) in the studied population.

**Figure 2:**
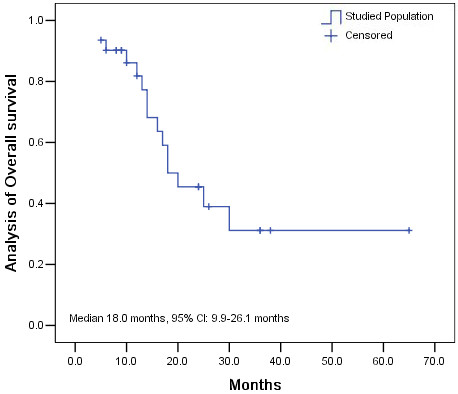
Analysis of overall survival (OS) in the studied population.

Follow-up studies continued until May 2012, with 5 patients lost to follow-up. The 1-year and 2-year survival rates were 83.3% and 42.8% respectively. The median PFS of all patients was 12.0 months (95% CI: 7.8–16.2 months) (Figure 1) and the median OS was 18.0 months (95% CI: 9.9–26.1 months) (Figure 2).

### Treatment-related toxicities

All the patients were evaluated for tretment-related toxicities (Table 3). The combination of IMRT and TP regimens were proved to be tolerable. The most common hematologic toxicity was neutropenia. Grade 3 neutropenia were observed in 5 (13.9%) patients. The non-hematological toxicities were generally found, but serious cases were relatively few. Grade 3 digestive tract side-effects, radiation esophagitis and radiodermatitis were observed in 4 patients (11.1%), 3 patients (8.3%) and 8 patients (22.2%) respectively. Nevertheless, it was important to note that two patients experienced treatment-related deaths for esophageal perforation and hemorrhea one month after CRT.

**Table 3:** Treatment-related toxicities

**Toxicities**	**Toxicity grades, n (%)**		
	**Grade 1**	**Grade 2**	**Grade 3**
***Hematological***			
Neutropenia	12(33.3)	8(22.2)	5(13.9)
Anemia	13(36.1)	5(13.9)	3(8.3)
Thrombocytopenia	10(27.8)	3(8.3)	3(8.3)
***Non-hematological***			
Digestive tract side-effects^*a*^	12(33.3)	5(13.9)	4(11.1)
Radiation esophagitis	7(19.4)	23(63.9)	3(8.3)
Radiodermatitis	3(8.3)	25(69.4)	8(22.2)

^*a*^: including nausea, vomiting and diarrhea.

## Discussion

In this study, for the first time, IMRT and concurrent TP regimen was demonstrated in patients of upper esophageal cancer, and had shown a promising activity. Our data showed that this strategy for patients with upper esophageal cancer produced clinical outcome, which was not worse than those results previously reported in esophageal cancer.

The concurrent CRT has been increasedly used as primary therapy regimen in patients who had unresectable esophageal carcinoma, were unwilling to undergo surgery, or were medically unfit for surgery. The RTOG 85–01 trial firstly analyzed the efficacy of CRT as a definitive treatment and revealed the superiority of CRT over radiotherapy alone in regards to 5-years overall survival [22]. Furthermore, Wong *et al.*[23] found that concomitant CRT is better than sequential CRT when a non-operative approach is selected for patients with localized esophageal cancer by meta-analysis. On these bases, several researchers investigated the optimal therapies strategies to prolong the survival and improve patient’s quality of life. A landmark study (INT 0123) found that combined-modality therapy consisting of 5-FU and DDP with concurrent 64.8 Gy was not better than the same regimen with concurrent 50.4 Gy in survival (13.0 *vs.* 18.1 months) and local/regional control (56% *vs.* 52%) [24]. In an attempt to improve these results, the RTOG 0113 phase II trial was designed to compare two different chemotherapy regimen including 5-FU, DDP and paclitaxel with concurrent 50.4 Gy of radiation in patients with localized esophageal cancer [25]. Although 5-FU-based group seems better than non-5-FU-based group and the result of INT 0123 study, it did not achieve the desired 1-year survival mark. Also, the two groups have 80% rate of grade 3 or 4 toxicities. Treatment-related death occurred in 3% and 6% of patients in two arms relatively. Therefore, neither of the two kinds of CRT strategies consisting of paclitaxel plus DDP proved to be sufficiently superior to the historical control of INT 0123 and warranted further investigation. In our study, the median OS was 18.0 months, which was relative higher than the results reported in TP-based group of the RTOG 0113 study (18 *vs.* 14.9 months). In addition, the grade 3 or 4 toxicities in our study were obviously lower than it, although the rates of the treatment-related death were similar (5.5% in our study and 6% in RTOG 0113 study). The reasons why this significant difference existed in two studies might be summarized as following: 1. the dose and time of TP regimen were different in two studies, and our regimen seemed more moderate; 2. 66% (23/35) of the patients in the TP-based CRT group of the 0113 trial were dignosed with the adenocarcinoma, while our patients were all squamous-cell esophageal carcinoma. The different pathological types might result in different response to CRT and the survival. However, our response rate was relatively low especially for CR rate when compared to those data reported previously. The possible reason might be the different chemotherapy schedule. In the previous studies, TP regimens were all scheduled weekly while ours was 3-week based plan. Moreover, surgery was performed in these studies that might have an impact on the results as well.

Most reported literatures about CRT of upper esophageal carcinoma have explored various combined-modality therapeutic schedules. Wang *et al.* reported significant results from a single institution experience of concurrent chemoradiation in 35 patients of cervical and upper thoracic esophageal cancer [17]. Median radiation dose was 50.4 Gy/28 fractions, and chemotherapy was 5-FU based. After a median follow-up of 39 months, the median PFS was 6 months and OS was 13 months. In addition, they showed that patients who received a radiation dose of greater than or equal to 50 Gy had a better outcome than those who received less than 50 Gy. In a recent study [18], the OS for patients in the up-front chemoradiation group was 24.9 months and the 2-year survival rate was 46.9%. The overall survival was very good probably because surgery followed CRT in 6 of 21 patients. Huang *et al.* reported their study compared the results of CRT based on 5-FU and either mitomycin C or DDP with 54 Gy of radiation with the high-dose DDP and 70 Gy of conformal radiation [19]. For all patients, the OS rate at 2 and 5 years were 46% and 28% in these patients treated curatively, respectively. However, no survival improvement could be showed after changing the treatment policy to high-dose cisplatin based and conventionally fractionated conformal radiotherapy.

To our knowledge, there were two studies investigated the TP regimen combining the conformal radiotherapy for esophageal carcinoma [26,27]. Both of them reported the weekly paclitaxel (intravenous infusion) and DDP with concurrent radiotherapy for esophageal carcinoma, some of these patients followed by surgery. Although the regimens were different between their studies and ours (3-week based), the outcomes still indicated that the TP regimen combined with radiotherapy for esophageal carcinoma was effective and tolerable.

In our study, all acute toxicities were tolerable (Table 3). The most common treatment-related toxicities included the radio-dermatitis and radiation-induced esophagitis. We had not observed the late-phase toxicities (such as pneumonitis, pleural effusion, and cardiac effusion), the reason might be that only partial volume of the lungs and heart had been irradiated during IMRT treatment. But one issue should be addressed here. In our study, two patients died because of esophageal perforation and hemorrhea after CRT. As reported in the RTOG 0113 trial, the majority of late radiation toxicities were related to esophageal injury [25]. We found that the huge ulcers (diameter ≥ 2 cm) were showed in the lesions of two patients. It seemed to suggest that those inevitable esophageal perforation and hemorrhea should be paid more attention in concurrent CRT for ulcerated carcinoma in practice.

In conclusion, our results showed that IMRT combined with concurrent TP regimen chemotherapy could be considered as an effective treatment with no significant toxicity in those patients with upper esophageal carcinoma. Currently, because all studies were small and retrospective, more studies on larger population are required to determine the specific treatment approach in upper esophageal carcinomas.

## Competing interests

The authors declare that they have no competing interests.
